# Campylobacter jejuni Enteritis Mimicking Mechanical Small Bowel Obstruction

**DOI:** 10.7759/cureus.103748

**Published:** 2026-02-16

**Authors:** Sina Shafiei, Atefeh Kalantary, Mohammad S Alghussein, Gholamreza Badiee, Amir H Talebi, Michael E Lazarus

**Affiliations:** 1 Internal Medicine, David Geffen School of Medicine at UCLA (University of California, Los Angeles), Los Angeles, USA; 2 Medicine, David Geffen School of Medicine at UCLA (University of California, Los Angeles), Los Angeles, USA; 3 Diagnostic Radiology, Los Robles Hospital and Medical Center, Thousand Oaks, USA

**Keywords:** abdominal pain, campylobacter jejuni, circumferential bowel edema, enteric pathogen panel, mechanical bowel obstruction, pseudo small bowel obstruction

## Abstract

In this case, we present an 84-year-old patient admitted for small bowel obstruction (SBO) with radiographic evidence of a clear transition point in the proximal small bowel on a computed tomography (CT) scan. Given his prior history of hemicolectomy, surgical adhesions were suspected as the likely cause. After several days of poor response to supportive treatment, he developed significant secretory diarrhea with stool samples positive for* Campylobacter jejuni.* In rare cases, *C. jejuni* enteritis can mimic SBO. We suggest that his SBO initially resembled mechanical obstruction on imaging due to significant bacterial induced circumferential bowel thickening and edema as opposed to the usual pseudo-obstruction from bacterial activated pro-inflammatory mediators, which disrupt intestinal motility. His obstruction resolved with a course of azithromycin antibiotics and conservative measures.

## Introduction

The most common clinical presentations of *Campylobacter* enteritis in adults include diarrhea, abdominal pain, and fever. Presentations can range from extreme abdominal pain with bloody stools and minimal or no fever to more typical gastroenteritis symptoms and signs. Most infections are self-limiting and do not require antibiotic treatment [1].

The pathophysiology of *Campylobacter jejuni-induced* pseudo-obstruction involves the bacterial activation of a sizable cascade of pro-inflammatory mediators that disrupt intestinal motility and cause direct damage to the intestinal epithelium. The current understanding suggests that the enteric nervous system is not directly affected. Altered bowel motility and eventually a significant secretory diarrhea are the manifestations of this process. The disrupted epithelial barrier can allow for bacterial translocation, which encourages further inflammation. When the inflammation leads to homogeneous circumferential thickening of the bowel wall, obstruction may occur [1]. CT scan imaging with oral and intravenous (IV) contrast can rarely reveal a discrete transition point mimicking true mechanical bowel obstruction. This imaging modality usually has a diagnostic accuracy that exceeds 90% for detecting mechanical small bowel obstruction (SBO) [2].

*C. jejuni *is a common cause of infectious diarrhea worldwide. About half of all patients with Campylobacteriosis report a history of bloody diarrhea [3]. It is highly unlikely to cause true mechanical SBO. These usually present as a true medical and surgical emergency. Fifteen percent of all emergency department (ED) visits for acute abdominal pain are due to an underlying SBO. These include mechanical and functional obstructions and usually present with constipation, obstipation, abdominal distention, nausea, vomiting, and abdominal pain. Medical management includes supportive care with bowel rest, hydration, and, in some cases, antibiotics. Surgery should be performed when conservative management fails, and this occurs in 20% of cases [4]. 

## Case presentation

An 84-year-old man with a past medical history of atrial fibrillation, aortic stenosis, hypertension, hyperlipidemia, and a remote history of right hemicolectomy for premalignant colonic polyps presented to the ED with epigastric pain, abdominal distention, nausea, and non-bloody vomiting for two days. He reported exertional dyspnea but denied any melena, chest pain, or urinary symptoms. He had no bowel movements for the prior three days but reported passing flatus. He denied any travel or sick contacts. His home medications included apixaban, 5 mg twice a day, diltiazem CD, 180 mg once a day, digoxin 0.125 mg once a day, rosuvastatin 10 mg once a day, and docusate 100 mg once a day as needed. He lived at home with his wife and denied any current or prior alcohol, tobacco, or illicit drug use.

In the ED, his blood pressure was 110/70 mmHg with an irregular heart rate of 77 beats per minute. He was oxygenating at 94% breathing ambient air, and his temperature was 36.6 C. Physical examination revealed mild distress due to abdominal distention and pain. He had no jugular venous distention. Cardiac examination showed irregular rhythm with a 2/6 crescendo-decrescendo systolic murmur, loudest in the second right intercostal space, consistent with mild aortic stenosis. Respiratory examination revealed normal bilateral breath sounds. His abdomen was distended with tenderness in the epigastrium but no rebound or guarding. His bowel sounds were hypoactive. His lower extremities were free of edema or cyanosis. His neurological examination showed no motor or sensory deficit.

Laboratory tests revealed a low hemoglobin with a normal white blood cell (WBC) and platelet count. His chemistry panel, including renal, liver function, and lactic acid were all within normal range. He had a normal anion gap. Review of his prior complete blood counts results showed that his anemia had progressed, and his hemoglobin had dropped from 14 g/dl three months prior.

**Figure 1:**
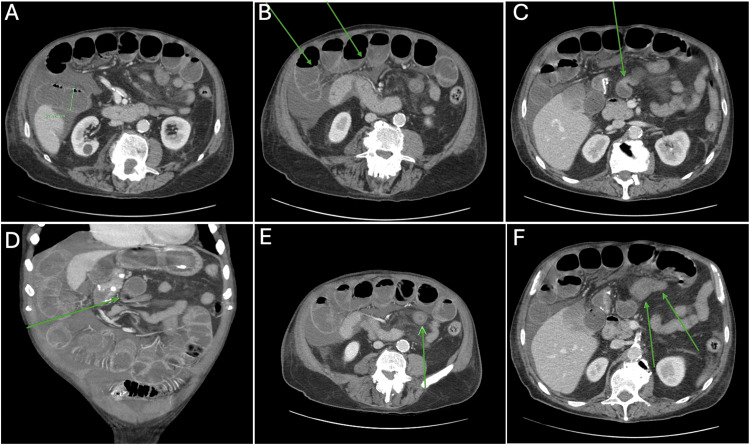
Abdominal CT images A and B: Prominent loops of dilated proximal small bowel with and without air fluid levels (arrows).  Small volume of ascites in the abdomen. No bowel pneumatosis, free air or frank pneumoperitoneum. C: Focal transition point in the central abdomen (arrow) on the axial view. D: Focal transition point in the central abdomen (arrow) on coronal view, distal to which the small bowel is decompressed. E and F: Short segment small bowel wall thickening distal to the transition point with accompanying surrounding mesenteric inflammatory changes and stranding (arrows).

A plain abdominal radiograph revealed dilated small bowel loops consistent with presumed SBO. A CT of his abdomen and pelvis without contrast demonstrated long segments of distended small bowel loops with air fluid levels (Figures 1A, 1B). A sharp transition point was identified in the central abdomen with no evidence of a mass (Figures 1C, 1D). A short segment of small bowel wall thickening distal to the transition point, with accompanying surrounding mesenteric inflammatory changes and stranding, was also observed. Based on appearance, findings favor inflammation rather than ischemia (Figures 1E, 1F). The CT scan also showed mild diffuse ascites. Although this finding was not initially felt to be associated with the bowel obstruction hypothesis, in retrospect it was likely related to the developing *Campylobacter* enteritis and associated focal peritoneal inflammation.

The initial appearance of a sharp transition point in the small bowel led to the hypothesis that a true mechanical obstruction was present and was supported by his history of prior surgery, with surgical adhesions being very high on our differential as the etiology. Nasogastric decompression and bowel rest (nil per os) were implemented. In addition, we performed serial abdominal examinations to monitor for progression and complications. Our workup of his anemia included stopping apixaban and excluding a hemolytic process. Hemolysis was excluded based on normal haptoglobin, lactate dehydrogenase, and unconjugated bilirubin levels (Table 1). Gastroenterology consultation was obtained in view of his new anemia. Esophago-gastro-duodenoscopy was performed to rule out upper gastrointestinal bleeding. This revealed a single gastric arteriovenous malformation (AVM), but no clear signs of recent bleeding were noted. The gastroenterologist cauterized his AVM appropriately.

**Table 1:** Laboratory Results The Enteric Pathogen Panel includes the followingmicro-organisms in addition to *Campylobacter Species and Clostridium Difficile: Salmonella, Shigella, ETEC-Enterotoxigenic Escherichia coli, EPEC Enteropathogenic Escherichia coli, EAEC- Enteroaggregative Escherichia coli, Shigatoxin E. coli, Vibrio Species, Yersinia enterocolytica, Giardia lamblia, Cryptosporidium, Entamoeba histolytica, Adenovirus F40/41, Noro Virus GI/GII, and Rota Virus,* all of which were negative

Laboratory Test	One Year Prior	Admission	Day 4	Normal Range
White Cell Count	-	6,400 cells/(µL)	15,000 cells/µL	4,000 to 11,000 cells/µL
Hemoglobin	14 g/dL	7.5 g/dL	7.2 g/dL	13.5-17.5 g/dL
Platelet Count	-	245,000/µL	-	150,000 to 450,000 /µL
Reticulocyte Count	-	2.4%	-	0.5-2.5%
Lactic Acid	-	1 mmol/L	-	0.5-1.5 mmol/L
C-Reactive Protein	-	1.27mg/L	-	< 3 mg/L
Haptoglobin	-	88 mg/dL	-	40 to 200 mg/dL
Unconjugated Bilirubin	-	0.4 mg/dL	-	0.2 to 0.8 mg/dL
Lactate Dehydrogenase (LDH)	-	117 U/L	-	100-280 U/L
Enteric Pathogen Panel	-	-	-	-
*Campylobacter* Species	-	-	Positive	Negative
Clostridium difficile PCR	-	-	Negative	Negative

Conservative treatment with nasogastric tube (NGT) decompression for three days resulted in no significant clinical improvement, and he continued to have output from his NGT (~200cc/day). The diagnostic momentum and imaging study suggesting a focal transition point of obstruction in a patient with prior abdominal surgery, coupled with our patient's poor response to conservative treatment and NGT decompression, led to a concern that his condition would progress without a definitive procedure to identify and lyse potential adhesions. A colorectal surgeon evaluated the patient and reviewed his imaging studies and recommended an exploratory laparotomy with a possible need for lysis of adhesions, given his prior history of hemicolectomy. While our patient was going through his preoperative optimization, he developed large volumes of bloody diarrhea multiple times per day, accompanied by worsening leukocytosis with a WBC count of 15,000 per microliter. Given the new onset of diarrhea and leukocytosis, stool samples were sent for Clostridium difficile toxin, polymerase chain reaction (PCR), and a gastrointestinal pathogen PCR panel, which was positive for *Campylobacter *on PCR. The patient was evaluated by an infectious disease specialist who recommended treating based on Infectious Diseases Society of America (IDSA) guidelines, which recommend considering antibiotics for *Campylobacter* enteritis in patients with particularly prolonged or severe disease, immunocompromised hosts, and those with bacteremia. Oral azithromycin 500 mg daily for three days was given. His symptoms improved, and surgical intervention was not needed. His NGT was removed. He resumed a regular diet and was discharged home in stable condition after a seven-day hospitalization. At day 17 post discharge, he remained asymptomatic with improved hemoglobin and normal white cell count.

## Discussion

Adhesive SBO involves physical luminal occlusion from adhesive bands (most commonly post-surgical adhesions) that mechanically block intestinal contents. Conversely, pseudo-obstruction results from intestinal hypoperistalsis or aperistalsis due to myogenic dysfunction. Causes include electrolyte disturbances, anticholinergic medications, and some intestinal infections. The bowel dilates because of impaired or absent bowel motility rather than a physical blockage. Both may present clinically with abdominal pain, constipation, and abdominal distention, and make definitive bedside differentiation a challenge. Adhesive SBO typically requires surgical intervention for complete obstruction, while pseudo-obstruction is managed supportively with bowel rest, correction of metabolic abnormalities, discontinuation of offending medications, and occasionally treating an underlying infectious process [5].

SBO is diagnosed with an abdominal CT scan with IV and oral contrast, which is highly sensitive and specific. A CT scan usually reveals the underlying cause. Post surgical adhesions were the most likely cause given his history and clinical presentation; however, mass lesions and bowel herniation were on the initial differential. The clinical presentation of SBO can range from asymptomatic to severe peritonitis with fulminant sepsis. It can lead to bowel perforation and acute abdomen when diagnosis and treatment are delayed. Fatality rates can be as high as 10% if it is not managed expeditiously [6]. Our patient presented with constipation, emesis, and a distended abdomen. His diarrhea presented late in the disease process and was caused by *C. jejuni *enteritis. *Campylobacter* is usually transmitted by infected poultry and their associated food products to humans [7]. Common risks for developing *C. jejuni* include international travel, use of proton pump inhibitors, and H2 inhibitors (histamine-2 receptor antagonists) [4]. The most common presenting symptoms are diarrhea and fever, followed by abdominal pain in over 90% of cases [5]. Typically, the diarrhea resolves after seven days, but the abdominal pain may persist [8]. In very rare cases, infection can be present with pancreatitis, SBO, and even cholecystitis. The cause of SBO can be due to electrolyte abnormalities and/or ongoing inflammation, which can cause homogenous circumferential thickening of the bowel wall, as likely occurred in our patient, seen in Figures 1E, 1F.

There are no reported cases in the medical literature of *Campylobacter*-associated bowel obstruction with a clear radiographic transition point. The available literature does not describe *Campylobacter* causing mechanical SBO. The one documented case involving both *Campylobacter* and bowel obstruction is a five-year-old child with concurrent *C. jejuni* bacteremia and intussusception [9]. However, intussusception represents a distinct pathophysiologic process where bowel telescopes into itself, rather than obstruction from adhesions or other mechanical causes, with a discrete transition point. In the pediatric literature, there is a reported association of increased risk of developing intussusception after an episode of infectious enteritis [9]. This has not been described in adults as far as we are aware.

In cases where patients fail to improve with conservative medical therapy, aggressive hydration, IV antibiotics, and surgical intervention may be needed, especially in the setting of bowel ischemia or bowel perforation. Should either of these complications arise, the mortality rate increases to 30% [10]. General clinical features that should prompt surgical intervention include diffuse peritonitis, ongoing fever, worsening leukocytosis, continuous pain, or metabolic acidosis. In our case, only one of these features was present: increased WCC. In addition, CT scan evidence of bowel ischemia or perforation are surgical emergency. Azithromycin is usually the first-line antimicrobial in symptomatic patients, but serious systemic infection should be treated with a parenteral aminoglycoside, such as gentamicin or imipenem [11].

## Conclusions

Adhesive SBO involves physical luminal occlusion from adhesive bands (most commonly post-surgical adhesions) that mechanically block intestinal contents. Conversely, pseudo-obstruction results from intestinal hypoperistalsis or aperistalsis due to peristaltic or myogenic dysfunction. Causes include electrolyte disturbances, anticholinergic medications, and some intestinal infections. The bowel dilates because of impaired or absent bowel motility rather than a physical blockage. Both may present clinically with abdominal pain, constipation, and abdominal distention and make definitive bedside differentiation a challenge. Adhesive SBO typically requires surgical intervention for complete obstruction, while pseudo-obstruction is managed supportively with bowel rest, correction of metabolic abnormalities, discontinuation of offending medications, and occasionally treating an underlying infectious process.

In this case report, we presented an atypical presentation of SBO in an elderly patient with risk factors for post-surgical adhesions and ischemic bowel who was admitted with symptoms of constipation, abdominal distention, and pain. He had no evidence of objective infection on presentation. Initial imaging revealed a transition point in the distal small bowel suggestive of mechanical obstruction. Furthermore, significant anemia on presentation further complicated the picture. Endoscopic evidence of an AVM in the gastric mucosa, hemodilution from IV fluids, and subsequent bloody diarrhea were the likely culprits. The delayed presentation of leukocytosis and *C. jejuni* dysentery with complete resolution after antimicrobial treatment highlights the atypical presentation and should inform clinicians to maintain vigilance for a broad differential, including intestinal pathogens, when managing patients with SBO. Our case can inform clinicians to avoid premature surgical intervention when infection remains a possible diagnosis. Based on our review of the literature, *Campylobacter* enteritis can present in many different ways, and classic infectious signs can manifest late in the clinical course.
